# Astragaloside IV Treats Parkinson's Disease by Regulating the Proliferation and Differentiation of NSCs through the SHH–Nurr1 Pathway

**DOI:** 10.1155/2024/2792909

**Published:** 2024-09-03

**Authors:** Zicong Wu, Jianing Zhang, Han Gao, Wentao Li

**Affiliations:** ^1^ Encephalopathy Department Shanghai Municipal Hospital of Traditional Chinese Medicine Shanghai University of Traditional Chinese Medicine, Shanghai 200071, China; ^2^ Fifth Medical Center of Chinese PLA General Hospital, Beijing 100071, China

## Abstract

Recently, there has been a surge of interest in enhancing the differentiation of neural stem cells (NSCs) and supplementing dopamine neurons as a potential treatment for Parkinson's disease, the second most prevalent neurodegenerative disorder. Two factors, sonic hedgehog (SHH) and nuclear receptor–related 1 protein (Nurr1), have been identified as influential in NSCs differentiation. Additionally, Astragaloside IV (AS-IV), an active compound derived from *Astragalus*, has also been discovered to impact NSCs differentiation. To assess the effects of AS-IV on cell activity, CCK-8 and flow cytometry techniques were employed. Meanwhile, western blotting, immunofluorescence, and real-time PCR were utilized to detect protein expression both *in vivo* and *in vitro*. Furthermore, siRNA assay was used to verify the association between SHH and Nurr1 and to investigate whether AS-IV exerts its effects through this pathway. The experimental findings revealed that AS-IV enhances cell activity and promotes the expression of differentiation proteins related to NSCs. Furthermore, the relationship between the SHH–Nurr1 pathway was confirmed, demonstrating that AS-IV induces NSCs differentiation via this pathway. Consequently, SHH, acting as the upstream signaling pathway of Nurr1, influences its expression, while AS-IV regulates the proliferation and differentiation of NSCs by modulating the SHH–Nurr1 pathway.

## 1. Introduction

Parkinson's disease (PD), ranking as the world's second most prevalent neurodegenerative disorder, emanates primarily from the progressive degeneration and depletion of dopaminergic neurons residing in the substantia nigra. Characterized by tremors, heightened muscular rigidity, and disturbances in autonomic nervous system function, these clinical manifestations have been widely recognized as key indicators for identifying PD [1]. The loss of dopamine neurons and the accumulation of misfolded *α*-synuclein within neurons are critical pathological features that play a significant role in the progression of PD [2, 3]. Hence, the principal approach to treating PD revolves around mitigating neuronal attrition and eradicating Lewy bodies.

Cell replacement therapy is an innovative cellular intervention approach that harnesses the remarkable potential of pluripotent stem cells to differentiate into different cell lineages. By utilizing cells to correct and replace damaged tissues or organs, the goal is to restore the harmonious balance of physiological function in the human body. The primary objective of using these multipotent stem cells in the field of disease treatment is to prevent, improve, or slow down the progression of the disease. Therefore, the application of cell therapy plays an increasingly important role in addressing the various challenges posed by PD [4]. Neural stem cells (NSCs) are responsible for producing neurons and glial cells in the brain and spinal cord. While most stem cells diminish during embryonic development in mammals, NSCs persist in certain areas of the adult brain [5]. By stimulating the differentiation of these NSCs, increasing the number of dopamine-producing neurons can be a potential treatment for PD.

Nuclear receptor–related 1 protein (Nurr1) was found in various regions of the nervous system, such as the cortex, hippocampus, spinal cord, and olfactory bulb [6]. Nurr1 is a crucial transcription factor essential for the growth, development, and maintenance of dopamine neurons. Research on Nurr1 has revealed that its expression in midbrain dopamine neurons diminishes with age, aligning closely with the onset of PD. This finding underscores the potential significance of Nurr1 in understanding and potentially targeting the mechanisms underlying PD [7]. Moreover, Nurr1 plays an anti-inflammatory role in the PD model by protecting neurons from inflammatory factors [8]. The sonic hedgehog (SHH) factor signaling pathway plays a critical role in embryonic development and later stages of life by regulating neuron proliferation and participating in essential physiological activities such as anti-inflammatory responses, oxidation, and autophagy [9]. Research has shown that SHH can induce the formation of dopamine neurons, when added to NSCs cultures *in vitro*, SHH can reactivate the functionality of Nurr1 in the brain, suggesting that SHH may serve as an important regulatory factor for Nurr1 [10].

Astragali radix, a traditional Chinese medicine, has been known to have therapeutic effects on various diseases. In particular, Astragaloside IV (AS-IV), the active ingredient of *Astragalus*, has shown significant effects in promoting the proliferation and differentiation of NSCs [11]. However, despite being recognized as one of the active ingredients of *Astragalus*, the specific impact of AS-IV on NSCs has not been fully elucidated. Therefore, this study aims to investigate the effects of AS-IV on the proliferation and differentiation of NSCs through the SHH–Nurr1 pathway, specifically in the context of treating PD.

## 2. Method

### 2.1. Animal

Male Wistar rats were obtained from Shanghai Slack Experimental Animals Co., Ltd. All rats were housed in a specific pathogen-free (SPF) animal room with controlled environmental conditions, including a temperature of 20−22°C and a humidity of 45%–65%. The rats were maintained on a 12-hr light/dark cycle and provided with standard feed throughout the study. The experimental rats were randomly divided into three groups: control group, model group, and AS-IV group, with 10 rats in each group. The PD rat model was established by stereotactic injection of 6-hydroxydopamine (6-OHDA) (#HY-B1081A, MedChemExpress, Monmouth Junction, NJ, USA). Rats in the AS-IV group received daily oral gavage of 30 mg/ml AS-IV [12] (#84687-43-4, Shanghai yuanye Bio-Technology Co., Ltd., Shanghai, China) after the model was established. All animal experiments were conducted in accordance with the National Institutes of Health Guide for the Care and Use of Laboratory Animals and approved by the Committee of the Shanghai Municipal Hospital of Traditional Chinese Medicine (#2019SHL-KYYS-04).

### 2.2. NSCs Extraction, Culture, and Identification

Selection of fetal rats from pregnant 14–17 Wistar rats (Tongxiang Branch of Zhejiang Weitonglihua Experimental Animal Technology Co., Ltd.). After the pregnant rats were executed by injection of an overdose of phenobarbital, the fetal rats were removed from the abdominal cavity of the pregnant rats, then, removing the brain of an embryonic rat, and the pure brain tissue obtained by stripping the brain of its meninges and vascular membranes. The brain tissue was collected using the 15 ml centrifuge tube. Brain tissue was collected in 15 ml centrifuge tubes, and precooled PBS solution was added. The tissue was dissociated by pasteurized tube, before cell suspension application via a 70-*μ*m cell strainer (#YA0943, Solarbio, Beijing, China).

After the cell suspension had settled, the supernatant was collected. The cells were then cultured in DMEM/F12 (#BL305A, Biosharp, Hefei, China) medium supplemented with B27 (10%; #17504044, Thermo Fisher Scientific, Cleveland, OH, USA), N-2 supplements (10%; #17502048, Thermo Fisher Scientific, Waltham, MA, USA), fibroblast growth factor (FGF-basic) (20 ng/ml; #400-29, Peprotech, Rocky Hill, NJ, USA), and epidermal growth factor (EGF) (20 ng/ml; #400-25, Peprotech, Rocky Hill, NJ, USA). The cells underwent two passages, and once neurospheres were formed, they were subjected to cell immunofluorescence staining for identification. The cells were plated on a confocal petri dish and fixed after 3 days with 4% paraformaldehyde. Following PBS wash, the cells were treated with 0.2% Triton X-100(#GC204003-100 ml, Servicebio, Wuhan, China) for 30 min and blocked with 2% BSA (#9048-46-8, Solarbio, Beijing, China). They were then incubated overnight at 4°C with an antibody specific to the NSCs marker protein nestin (1 : 250; #DF7754, Affinity, Jiangsu, China), followed by incubation with goat anti-rabbit IgG (H + L) FITC (1 : 500; #S0008, Affinity, Jiangsu, China). Finally, the slides were sealed with a antifade mounting medium with DAPI (#P0131-5 ml, Beyotime, Shanghai, China) and observation under a confocal microscope (#SP8, Leica, Wetzlar, Germany).

### 2.3. Flow Cytometry

The apoptosis rate of NSCs with AS-IV intervention was analyzed using flow cytometry. NSCs cells were seeded on a 6-well plate and cultured for 3, 7, and 14 days. The cells were then washed with cooled PBS and stained using the Annexin V-FITC apoptosis detection kit (#C1062S, Beyotime, Shanghai, China). Finally, the stained cells were analyzed using a flow cytometer (CytoFLEX; Beckman Coulter, Brea, CA, USA) to measure the apoptosis rate.

### 2.4. CCK-8

NSCs (5 × 10^4^/100 *μ*l) were inoculated into each well of a 96-well plate and cultured overnight. AS-IV was dissolved in DMSO (#ST038-100 ml, Beyotime, Shanghai, China), and different concentration gradients were set to intervene with the cells for 24 hr. The concentration that showed the optimal promotion of cell viability was selected. Subsequently, the NSCs were intervened with the selected concentration for 24, 48, and 72 hr, respectively, and cell viability was measured using cell counting kit-8 (CCK-8) (#BEB22001, BEB, Nanjing, China). According to the manufacturer's instructions, use an enzyme marker to calculate cell viability at 450 nm.

### 2.5. Western Blotting

NSCs were inoculated into 6-well petri dishes and incubated for 3, 7, and 14 days. After that, RIPA lysate containing a mixture of PMSF and phosphatase inhibitor was added, the supernatant was collected after a 30-min incubation. After inducing rats' death with an overdose of anesthetics, the striatum of rats was extracted. The brain tissue suspension was obtained using ultrasonic lysis method, and the total protein of the striatal tissue was extracted using the same method as cell protein extraction. Next, the total protein was quantified using the enhanced BCA protein assay kit (#P0010, Beyotime, Shanghai, China). Proteins were electrophoresed on SDS-PAGE for 1 hr. Subsequently, a constant current transfer membrane at 300 mA was used for 1.5 hr to transfer the proteins onto a PVDF membrane. After blocking the PVDF membrane with skim milk powder, the primary antibodies, including Gapdh (1 : 1000; #GB15004-100, Servicebio, Wuhan, China), proliferative cell nuclear antigen (PCNA) (1 : 1000; #AF0239, Affinity, Jiangsu, China), class III beta-tubulin (Tuj-1) (1 : 1000; #T2200-200UL, Sigma–Aldrich, St Louis, USA), tyrosine hydroxylase (TH) (1 : 1000; #AF6113, Affinity, Jiangsu, China), SHH (1 : 1000; # BA2171, BOSTER, Wuhan, China), and Nurr1 (1 : 1000; #DF12678, Affinity, Jiangsu, China), were incubated overnight at 4°C. The secondary antibody, goat anti-rabbit (1 : 1000; #RGAR001, Proteintech Group, Wuhan, China), was then incubated for 1 hr. Finally, the BeyoECL star (#P0018AM, Beyotime, Shanghai, China) was used, and data collection was performed using a fully automated chemiluminescence imaging analysis system (Tanon-5200, Shanghai Tianeng Technology Co., Ltd., China).

### 2.6. Immunofluorescence

The original NSCs were evenly spread on a confocal petri dish, cultured with AS-IV (2 *µ*g/ml) for 3, 7, and 14 days, 4% polyoxymethylene fixed for 30 min, PBS cleaned, and TritonX-100 (#GC204003-100 ml, Servicebio, Wuhan, China) diluted with PBS-T to 0.2%, treated for 25 min, 5% BSA (#9048-46-8, Solarbio, Beijing, China) closed for 30 min, dopamine transporter (DAT) (1 : 200; #ab221845, Abcam, Cambridge, UK), Tuj-1 (1 : 200; #T2200-200UL, Sigma–Aldrich, St Louis, USA) incubated overnight at 4°C, the next day use goat anti-rabbit IgG (H + L) FITC (1 : 500; #RGAR002, Proteintech Group, Wuhan, China), goat anti-mouse IgG (H + L) Fluor594 (1 : 500; #RGAM004, Proteintech Group, Wuhan, China) combined for 1 hr, used antifade mounting medium with DAPI (#P0131-5 ml, Beyotime, Shanghai, China), and finally observed under a confocal microscope (#SP8, Leica, Wetzlar, Germany). After the paraffin-embedded tissue sample is sliced, xylene and alcohol gradient dewaxing and rehydration, PBS cleaning, H_2_O_2_ and trypsin are treated separately for 30 min, fixed with 4% polyoxymethylene, transparent by TritonX-100, BSA closed, PCNA (1 : 200; #AF0239, Affinity, Jiangsu, China), Tuj-1 (1 : 200; #T2200-200UL, Sigma–Aldrich, St Louis, USA), TH (1 : 200; #AF6113, Affinity, Jiangsu, China), GFAP (1 : 250; # DF6040, Affinity, Jiangsu, China), caspase-3 (1 : 500; #9664, Cell Signaling Technology, Massachusetts, USA) 4°C overnight. It was then cointeracted with goat anti-rabbit IgG (H + L) FITC (1 : 500; #RGAR002, Proteintech Group, Wuhan, China) for 1 hr using the antifade mounting medium with DAPI, and finally visualized under a confocal microscope (#SP8, Leica, Wetzlar, Germany), and fluorescence intensity was counted by ImageJ.

### 2.7. Real-Time Quantitative PCR

According to the instructions specified by the manufacturer, total RNA was extracted from NSCs and animal tissue using Trizol reagent (#10296010, Thermo Fisher Scientific, Waltham, MA, USA) to detect TH, Tuj-1, and PCNA expression, and an equal amount of total RNA (0.5 *μ*g) was reverse transcribed to cDNA and qRT-PCR (#RR036A, Takara, Dalian, China) by SYBR green I real-time fluorescence quantitative PCR, RT primer design (Table 1), and then real-time fluorescence quantitative PCR using the real-time fluorescence quantitative PCR system (#ABI7300; Applied Biosystems Inc., USA). The 2^−*ΔΔ*Ct^ method to calculate the relative gene expression levels of DAT, Tuj-1, and PCNA with Gapdh and *β*-actin as parameters.

### 2.8. siRNA Transfection

The NSCs were placed in a 6-well plate for culture, SHH/Nurr1-siRNA primers were designed, synthesized by RiboBio Biotechnology Co., Ltd., and siRNA kit (RiboBio, Guangzhou, China) was used to intervene with a transfection concentration of 50 nM per well. Dilute the siRNA, dilute 5 *µ*l-20 *µ*M siRNA storage solution with 120 *µ*l 1X ribo FECT™P buffer per well, mix gently, add 12 *µ*l ribo FECT™P reagent (RiboBio, Guangzhou, China), gently blow and mix well, incubate at room temperature for 15 min to prepare a transfection complex, and then add the ribo FECT™P transfection complex to an appropriate amount of nonantibody complete medium and mix gently. Finally, the 6-well plate was placed in a cell culture incubator for 48 hr of transfection.

### 2.9. Behavioral Detection

The behavioral changes of each group of rats were assessed using the circle experiment and the balance beam experiment to evaluate the therapeutic effect of AS-IV on PD rats.

In the circle test, the rats were subcutaneously administered 0.2 mg/kg apomorphine hydrochloride (#41372-20-7, Sigma–Aldrich, St Louis, USA) dissolved in 0.1% ascorbate saline solution. The success of the model was determined if the number of rotations to the healthy side exceeded seven rotations per minute.

For the balance beam test, the rats were placed on a wooden bar with a width of 1.5 cm and a length of 100 cm. The height of the wooden bar from the ground was 20 cm, and the two ends were fixed to a 10 cm × 10 cm flat plate. Prior to modeling, each group of rats underwent training to walk on the balance beam. Normal rats were able to walk on the balance beam for 2 min without falling. After three training sessions, the rats were able to walk on the balance beam without any issues.

Once the model was established and drug treatment was administered, the balance of each group of rats was evaluated and given corresponding scores as follows:

1. Score 0: The rat jumps on the balance beam and walks without falling.
2. Score 0−1: The rat jumps on the balance beam, and there is a chance of falling that is less than 50% while walking.
3. Score 1−2: The rat jumps on the balance beam, and there is a chance of falling that is greater than 50% while walking.
4. Score 2−3: The rat jumps on the balance beam with the assistance of the healthy hind limbs, but the affected hind limbs cannot help with forward movement.
5. Score 3−4: The rat cannot walk on the balance beam but can sit on it.
6. Score 4−5: When placed on the balance beam, the rat will fall down.

### 2.10. Statistics

Statistical analyses were performed using SPSS Grader Pack Version 21.0 statistical software (SPSS, Inc., Chicago, USA). All data are expressed as mean ± SEM or mean ± SD. Differences between the two groups were analyzed using Student's t-test, and heterogeneity between groups was analyzed using one-way analysis of variance (ANOVA). *P* < 0.05 were considered to be statistically significant.

## 3. Results

### 3.1. NSCs Identification and Activity Detection

**Figure 1:**
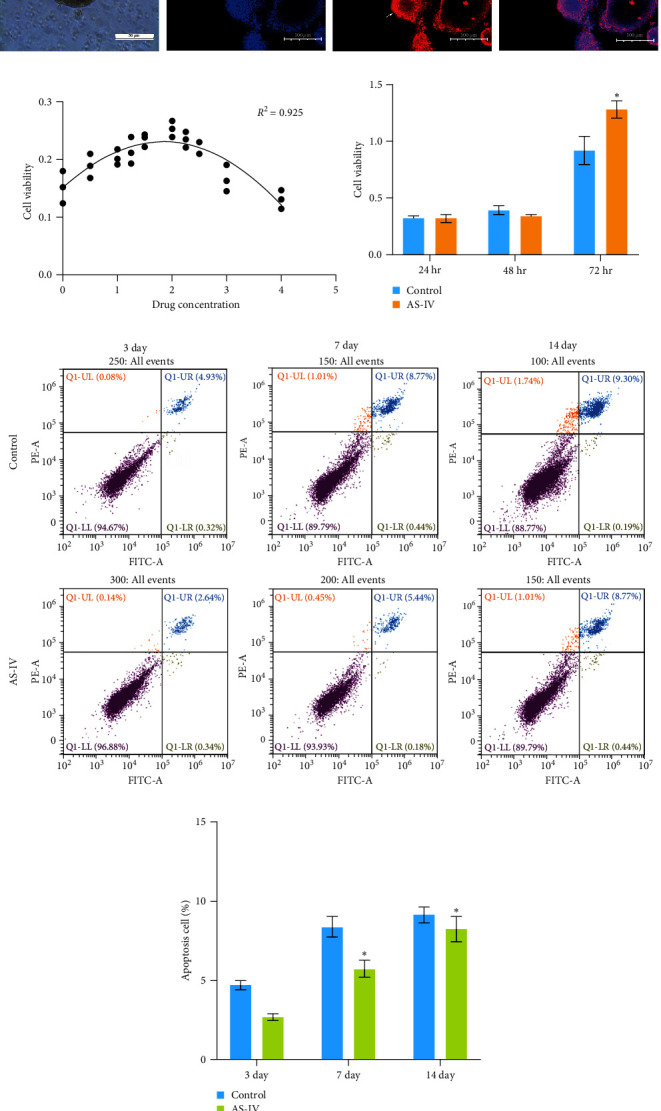
AS-IV promotes activity and inhibits apoptosis in NSCs: (a) the cells were observed to form neurospheres, as indicated by the arrows in the image (scale bar = 100 *μ*m). The third generation of cells was identified as NSCs by treating them with DAPI (blue) and Nestin (red) staining (scale bar = 100 *μ*m); (b) the effect of different concentrations of AS-IV on cell activity was screened using the CCK-8 assay. The *x*-axis represents the drug concentration, and the *y*-axis represents the cell activity. The coefficient of determination (*R*^2^ = 0.925) indicates a good fit of the data; (c) cell activity was assessed using the CCK-8 assay at 24, 48, and 72 hr after treatment with AS-IV; (d) flow cytometry was used to detect the apoptosis rate of NSCs after 3, 7, and 14 days of AS-IV intervention; (e) the results of flow cytometry were statistically analyzed. The data are presented as mean ± SEM, with *n* ≥ 3. ( ^*∗*^*P* < 0.05 vs. control group).

The proliferation and activity of NSCs are closely linked to the number and distribution of neurons in the brain. When neurons in the brain die, NSCs can utilize their differentiation potential to partially replenish the lost neurons. In this study, embryonic-derived NSCs were obtained and cultured through successive generations. Immunofluorescence staining was performed to identify the third-generation cells (Figure 1(a)). After confirming the cells as NSCs through nestin staining, they were subjected to CCK-8 cell activity detection in a 96-well plate, we screened the optimal concentration of AS-IV by adding different concentrations of the drug to intervene in the cells, and the results showed that 2 *μ*g/ml of AS-IV had the greatest effect on cell activity (Figure 1(b)). Subsequently, by comparing the effect of AS-IV intervention on cell activity after 24, 48, and 72 hr, a statistically significant difference in the promotion of cell activity was observed after 72 hr of AS-IV intervention (Figure 1(c)). Consequently, the cells were transferred to a 6-well plate for long-term AS-IV intervention. Flow cytometry results indicated that AS-IV could inhibited the apoptosis of NSCs (Figures 1(d) and 1(e)). These findings demonstrated the effective promotion of NSCs activity and inhibition of apoptosis by AS-IV.

### 3.2. Protein and mRNA Expression after AS-IV Intervention in NSCs

**Figure 2:**
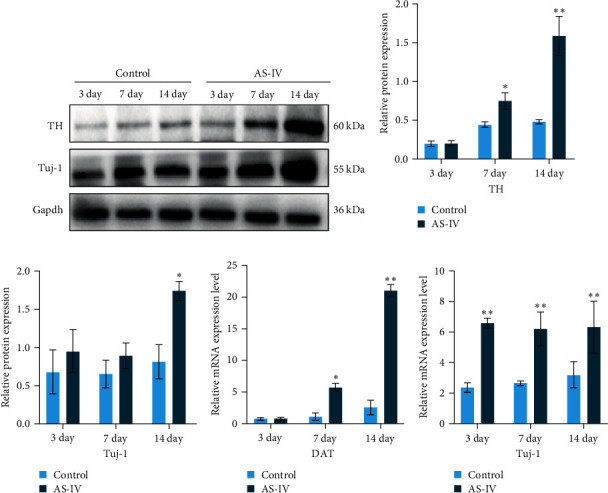
The images demonstrate the effect of AS-IV on NSCs: (a) western blotting detected protein expression in AS-IV interfused NSCs at 3, 7, and 14 days; (b) grey values of TH proteins were calculated using ImageJ, and statistical differences were analyzed using SPSS one-way ANOVA; (c) Tuj-1 protein was calculated using ImageJ with grey values; (d) qRT-PCR data of TH and statistical differences; (e) qRT-PCR data of Tuj-1 and statistical differences. Statistical differences were analyzed by ANOVA; data were expressed as mean ± SEM; *n* ≥ 3, ( ^*∗*^*P* < 0.05 vs. control group and  ^*∗∗*^*P* < 0.005 vs. control group).

The expression levels of TH and tuj-1 were significantly enhanced in the AS-IV group compared to the control group on days 7 and 14 after AS-IV-induced differentiation. Western blotting was performed to detect protein expression (Figure 2(a)). The expression of TH protein in the AS-IV group was significantly different from the control group after 7 days of intervention, and the difference became even more significant after 14 days (Figure 2(b)). Furthermore, qRT-PCR data showed that AS-IV intervention also promoted DAT transcription on day 7, which was consistent with the western blotting results for TH protein levels (Figure 2(c)). Interestingly, qRT-PCR testing for Tuj-1 demonstrated significant differences in the AS-IV group from the 3^rd^ day, although it took 14 days before a statistical difference in protein expression was observed compared to the control group (Figure 2(d)). The results demonstrated that intervention with AS-IV can promote the expression of TH, DAT, and Tuj-1, suggesting that AS-IV can induce the transformation of NSCs into dopamine neurons.

### 3.3. Immunofluorescence Detection of NSCs Differentiation and Fluorescence Intensity

**Figure 3:**
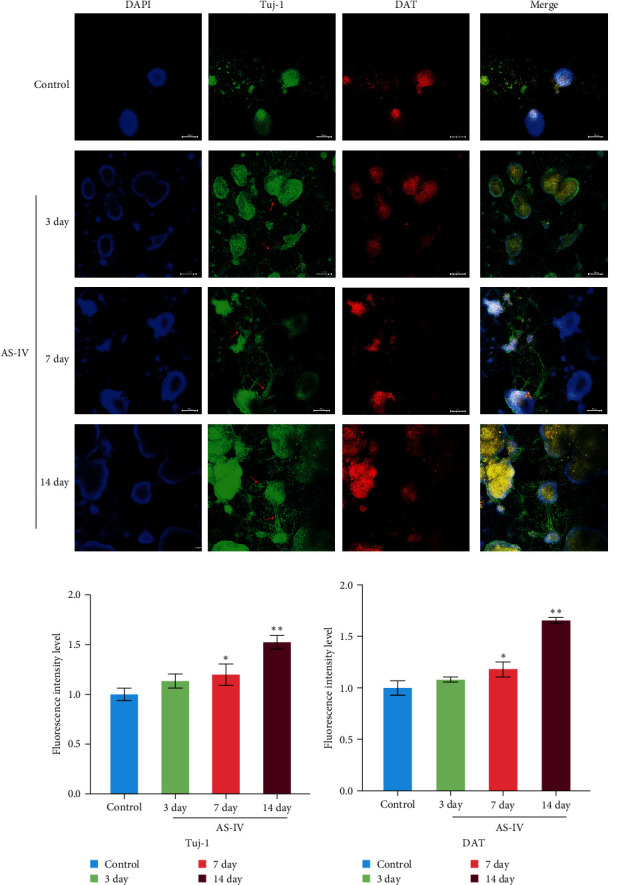
The fluorescence intensity of different protein expression was counted after 3, 7 and 14 days of AS-IV intervention: (a) expression of DAT (red) and Tuj-1 (green) after treating with AS-IV for 3, 7, and 14 days; DAPI was used to counterstain nuclei (scale bar = 100 *μ*m); (b) quantitative analysis of relative Tuj-1 positive cells; (c) fluorescence intensity analysis of protein expression of DAT in NSCs cells. The data are expressed as mean ± SD; *n* ≥ 3, ( ^*∗*^*P* < 0.05 vs. control group and  ^*∗∗*^*P* < 0.005 vs. control group).

We observed that in the absence of external intervention, NSCs underwent slow differentiation over a 14-day period. To assess the impact of AS-IV on NSC differentiation, immunofluorescence was used to evaluate the expression of DAT and Tuj-1 after AS-IV intervention, followed by quantification of fluorescence intensity using ImageJ (Figure 3). Subsequently, three time periods within the 14-day window were selected for testing based on previous culture methods. When fluorescence double staining was performed to evaluate cell differentiation, it was observed that after 3 days of AS-IV intervention, there was an increase in cell number as well as expression of DAT and Tuj-1 proteins in NSCs. Notably, Tuj-1 started to differentiate and formed tentacle-like structures (Figure 3(a)). Compared to the control group without AS-IV intervention at day 7, statistically significant differences were found in DAT fluorescence intensity (Figure 3(b)) and Tuj-1 fluorescence intensity (Figure 3(c)), after AS-IV intervention in NSCs. Moreover, after 14 days of continuous AS-IV intervention in NSCs, the difference in fluorescence levels became even more significant. These results are consistent with earlier findings obtained from protein expression analysis using western blotting and mRNA detection.

### 3.4. Detection of Protein Expression after siRNA Transfection of Cells

**Figure 4:**
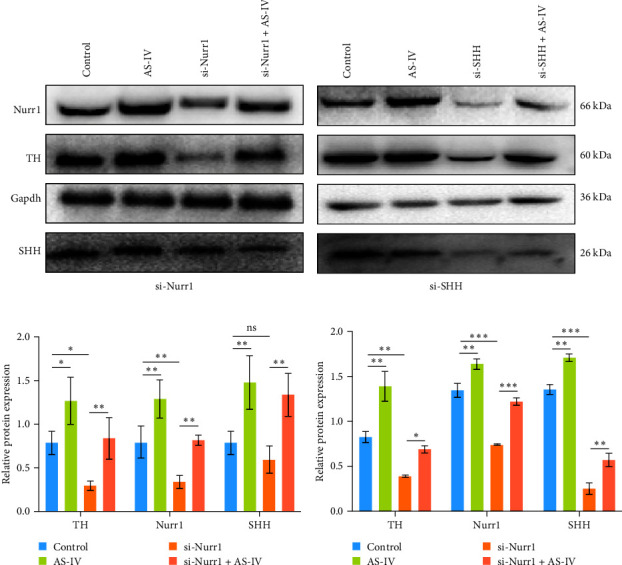
siRNA interferes with intracellular protein expression: (a) and (b) western blotting for protein expression of Nurr1, TH, and SHH after transfection of si-Nurr1 and si-SHH; (c) and (d) One-way ANOVA statistics of the data using SPSS; data are expressed as mean ± SEM; *n* ≥ 3, ( ^*∗*^*P* < 0.05,  ^*∗∗*^*P* < 0.005 vs. control group, and  ^*∗∗∗*^*P* < 0.001 vs. control group).

To ascertain the involvement of AS-IV in the SHH–Nurr1 pathway and to confirm the sequence of events within the pathway, cells were transfected with Si-Nurr1 and Si-SHH, respectively. Following the intervention of AS-IV, western blotting analysis was performed to assess the expression of relevant proteins (Figures 4(a) and 4(b)). This approach served to verify if AS-IV modulates the differentiation of NSCs into dopamine neurons via the SHH–Nurr1 pathway, as indicated by alterations in protein expression levels. The result revealed that AS-IV enhances the expression of Nurr1 (Figure 4(c)) by upregulating the levels of SHH protein (Figure 4(d)), consequently leading to increased expression of TH protein (Figures 4(c) and 4(d)).

### 3.5. Behavior Test

**Figure 5:**
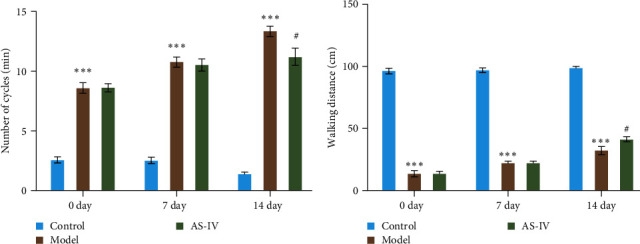
Behavioral performance of PD model rats treated with AS-IV: (a) the number of cycles by the rats and (b) the distance moved on the balance beam; the data are expressed as mean ± SD; *n* ≥ 3, ( ^*∗∗∗*^*P* < 0.001 vs. control group and #*P* < 0.05 vs. model group).

The therapeutic effect of AS-IV on rats with PD was assessed by comparing the behavior of rats in each group through the spinning circle experiment and the balance beam experiment. In the rat circling experiment, the results showed that the AS-IV group had a downward trend in the number of circulations compared to the model group after 7 days of modeling, but there was no significant difference; after 14 days of modeling, the number of circulations of rats in the *Astragalus* AS-IV group was lower than that of the model group (Figure 5(a)). In the balance beam test, PD rats had a short retention time on the balance beam and unable to reach the destination. After 7 days of modeling, there was no significant difference between the AS-IV group and the model group; after 14 days of modeling, the retention time in the balance beam of rats in the AS-IV group was slightly longer than that of the model group, and the scores of the model group and the AS-IV group were significantly different (Figure 5(b)).

### 3.6. Immunofluorescence Detection of Dopaminergic Cell Expression in the Brain

**Figure 6:**
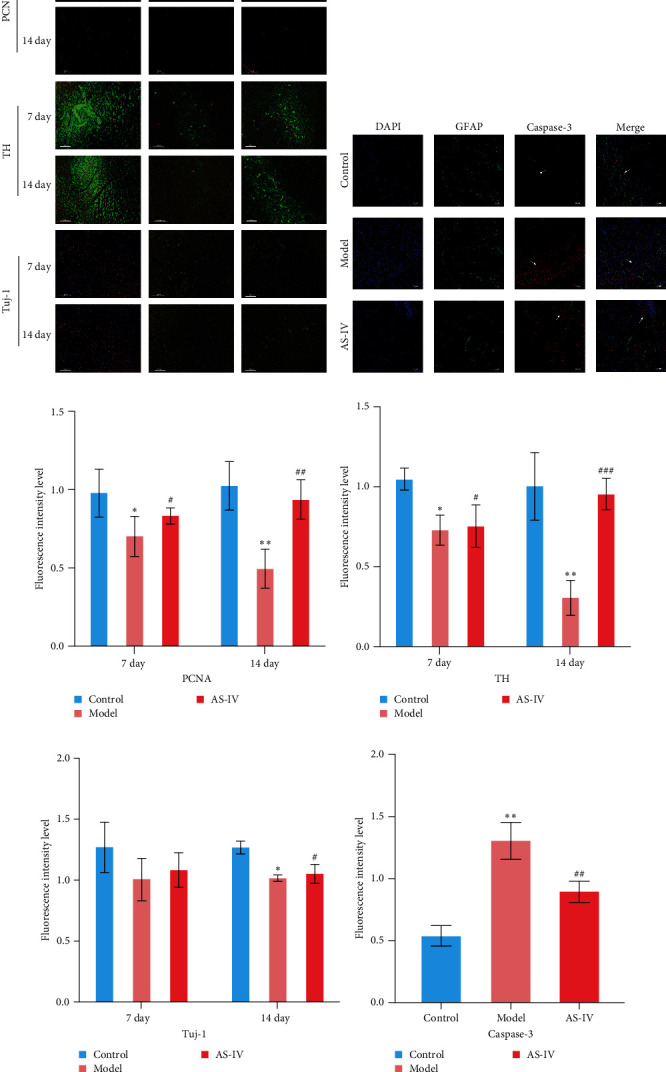
Immunofluorescence was employed to detect protein expression in rat brain tissue, followed by statistical analysis: (a) brain sections treated with DAPI (blue), PCNA (red), TH (green), Tuj-1 (orange), 7 and 14 days after AS-IV intervention, observed under a 10x microscope (scale bar = 100 *μ*m); (b) staining of the brain sections with DAPI (blue), GFAP (green), caspase-3 (red) (scale bar = 100 *μ*m), arrows represent caspase-3 protein fluorescence; (c) statistical comparison of fluorescence intensities of PCNA by the independent samples *t* test for PCNA; (d) fluorescence intensity of TH by independent samples *t* test; (e) fluorescence intensity of PCNA by independent samples *t* test; (f) fluorescence intensity of caspase-3 by independent samples *t* test; the data are expressed as mean ± SEM; *n* ≥ 3, ( ^*∗*^*P* < 0.05 vs. control group,  ^*∗∗*^*P* < 0.005 vs. control group, #*P* < 0.05 vs. model group, ##*P* < 0.005 vs. model group, and ###*P* < 0.001 vs. model group).

Immunofluorescence was used to detect the expression of dopaminergic neurons in the rat brain (Figure 6) and the percentage of apoptotic neurons in the brain after 14 days. A horizontal comparison was made between the fluorescence intensity levels of various protein expressions in the brain at day 7 and day 14. (Figure 6(a)) revealed that the PCNA fluorescence intensity levels in the AS-IV group were altered compared with the model group at the 7^th^ day, and this difference was further increased after 14 days (Figure 6(c)). After the AS-IV treatment for 7 days, the TH levels were significantly higher compared with the model group, accompanied by the prolongation of AS-IV intervention time, the level of TH protein also gradually to return to normal (Figure 6(d)). Tuj-1 was detected by fluorescence intensity test and found that there was significant difference between the AS-IV group and the model group within 14 days (Figure 6(e)). The level of neuronal apoptosis in the brain of AS-IV-treated PD model rats was evaluated by detecting the expression level of caspase-3 after 14 days (Figure 6(b)), and the results showed that the AS-IV treatment after 14 days, the expression of caspase-3 decreased dramatically (Figure 6(f)), which indicated that neuronal cell death within the brain of PD rats was suppressed.

### 3.7. Expression of Dopamine Cell Protein and mRNA in Rats with AS-IV Intragastric Administration

**Figure 7:**
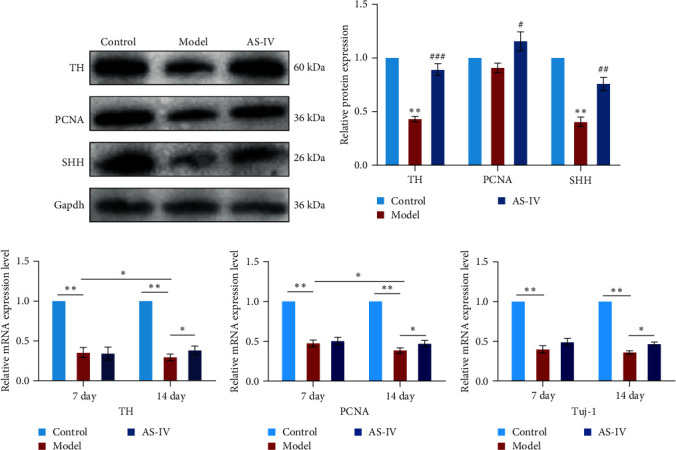
Protein changes in the rat brain were investigated and subjected to statistical analysis. (a) protein expression in the rat's brain 14 days after modeling; (b) TH, PCNA, and SHH protein expression; (c) TH mRNA expression level in the brain; (d) the TH PCNA mRNA expression level in the brain; (e) Tuj-1 mRNA expression level in the brain; The data are expressed as mean ± SEM; *n* ≥ 3, ( ^*∗*^*P* < 0.05 vs. control group,  ^*∗∗*^*P* < 0.005 vs. control group, #*P* < 0.05 vs. model group, ##*P* < 0.005 vs. model group, and ###*P* < 0.001 vs. model group).

Seven days after modeling, the expression of PCNA, Tuj-1, and TH mRNA in the rat brain decreased significantly. After 14 days, the expression of PCNA, Tuj-1, and TH mRNA continued to decrease compared with 7 days, and PCNA and TH decreased significantly (Figures 7(c), 7(d), and 7(e)). Fourteen days after AS-IV treatment, the expression of PCNA, Tuj-1, and TH mRNA increased significantly compared with the model group, proving that after 14 days of AS-IV intervention, the treatment effect was significant (Figures 7(c), 7(d), and 7(e)).Therefore, the striatum of PD rats with AS-IV intervention for 14 days was quantitatively tested for western blotting (Figure 7(a)). Compared with the model group, the expression of SHH, PCNA, and TH protein in the AS-IV group was significantly different and statistically significant (Figure 7(b)), which were consistent with the mRNA test results.

## 4. Discussion

This experimental study demonstrated the impact of AS-IV on NSCs' proliferation and differentiation. The results indicated that AS-IV boosts NSCs activity, decreases apoptosis, and triggers NSCs to differentiate into dopamine neurons via the SHH–Nurr1 pathway.

The main characteristics of NSCs include their potential to differentiate into other types of neurons and their persistent capacity for self-renewal. NSCs primarily remain in a dormant state and serve as a cellular reservoir for brain neurons. They only undergo renewal to become other tissues or neuronal cells during specific moments or when stimulated by external factors [13]. AS-IV intervention in NSCs significantly reduced apoptosis and increased cell viability of NSCs, and this effect was more pronounced with longer intervention time.

Tuj-1 is mainly composed of microtubule proteins and is found in the cell bodies, axons, and dendrites of neurons. As an early marker protein for neuronal differentiation, it has been widely confirmed to specifically identify neuronal class III *β*-tubulin in neuronal cells, but not *β*-tubulin in glial cells. The expression level of Tuj-1 often indicates the extent of cellular differentiation in the central nervous system. Therefore, it is common to detect Tuj-1 to observe the specificity of neural stem cell differentiation [14, 15, 16]. The study utilized AS-IV *in vitro* to induce the differentiation of NSCs. The results revealed that AS-IV exhibited an early effect on Tuj-1 expression at days 3, 7, and 14, and following 7–14 days of intervention, NSCs were found to differentiate into dopamine neurons. Subsequently, in the Wistar rat model, AS-IV intervention reflected a similar trend in the expression of Tuj-1 in the brain. Additionally, the study also examined the expression of the dopamine neuron marker DAT and TH. DAT, an essential protein for dopaminergic neurons, regulates dopamine transport and cell-to-cell homeostasis. It plays a pivotal role in dopamine clearance within the striatum, representing ~80% of this region [17].

TH and DAT are closely linked in their involvement in the pathology of PD. TH, an enzyme that catalyzes the first step in dopamine synthesis by converting tyrosine into dopamine, is also a notable marker of dopaminergic neurons. Conversely, DAT is primarily located on neuron cell membranes and is responsible for the transportation of dopamine to synapses, where it plays a crucial role in neurotransmitter transmission. Notably, the lack of dopamine in the basal ganglia is a fundamental contributor to the motor dysfunction observed in PD. Research has identified a correlation between the accumulation of *α*-synuclein and other forms of neural decline and a decrease in total protein content of TH, indicating that TH is not only a marker protein and speed-limiting enzyme but also profoundly implicated in the pathogenesis and progression of PD [18]. Alterations in levels of TH and DAT are often indicative of the onset and progression of PD and can impact the motor function of affected individuals. In our investigation, we aimed to observe the effects of AS-IV treatment on the levels of DAT and TH both *in vivo* and *in vitro*. Our findings demonstrated that AS-IV effectively increased the expression levels of both proteins. Additionally, behavioral tests conducted on PD model rats revealed that AS-IV treatment significantly enhanced their mobility and balance compared to the untreated model group. These results strongly suggest that AS-IV improves motor function in PD by promoting the expression of Tuj-1, DAT, and TH.

After AS-IV treated the PD model for 7 and 14 days, we examined the changes in PCNA, a nuclear protein closely associated with DNA synthesis, repair, and translation [19], as an indicator of dopaminergic neuron proliferation in the substantia nigra striatum. An increase in PCNA levels is indicative of active cell proliferation. Our experimental result demonstrated that AS-IV effectively enhanced the expression of PCNA in dopaminergic cells within the striatum of the substantia nigra. This observation suggests that AS-IV has the ability to reactivate degenerated dopamine neurons and augment their quantity. Given the widely accepted notion of a window period for the proliferation of dopaminergic neurons and the limited proliferation of adult dopaminergic neurons, we suggest that AS-IV may induce the differentiation of NSCs in the brain, thus replenishing the deficient neurons in the nigra striatum.

SHH protein plays a crucial role in directing the development of the central nervous system, limbs, face, and other organs. It is involved in regulating cell proliferation, cell differentiation, and organizational structure. Additionally, during the developmental process of the central nervous system, SHH, and fibroblast growth factor 8 (FGF8) function as diffusible factors. Their interaction leads to the induction of neuronal differentiation in the ventral side of the midbrain [20]. Previous research has shown that the SHH signaling pathway can work together with other pathways to encourage the differentiation of omnipotent stem cells into dopaminergic neurons [21], it also guides the transformation of these precursor cells into functional dopaminergic cells that highly express Nurr1 and TH [22]. Nurr1, a key transcription factor for the development of dopaminergic neurons, is closely associated with synuclein in these neurons. Notably, the overexpression of *α*-synuclein has been found to result in diminished transcription of Nurr1. Conversely, Nurr1 has been shown to exert a substantial inhibitory effect on *α*-synuclein transcription [23, 24]. This reciprocal regulation between Nurr1 and *α*-synuclein suggests a bidirectional relationship that may influence the onset and progression of PD. Given that PD is characterized by neuronal degeneration, the protective role of Nurr1 in dopaminergic neurons is of crucial importance. This entails the potential for SHH to regulate the expression of Nurr1 as part of the upstream signaling pathway, with AS-IV influencing this pathway. Consequently, we utilized siRNA to individually suppress the expression of SHH and Nurr1, with the experimental outcomes aligning with our anticipated results. Our findings indicated that AS-IV impacts the level of Nurr1 by upregulating the expression of SHH, leading to increased levels of TH and DAT, and prompting the directed differentiation of NSCs into dopaminergic neurons.

AS-IV can effectively improve the behavior of PD rat models, promote the proliferation of NSCs, and induce the directional differentiation of NSCs into dopaminergic neurons through the SHH–Nurr1 pathway. This mechanism also involves the upregulation of the expression of TH and DAT in the neuronal cells. In contrast, the current clinical drugs primarily focus on increasing the half-life of dopamine and delaying corresponding symptoms of PD, lacking a targeted treatment approach for the disease. This study not only furnishes corroborative evidence for the therapeutic potential of AS-IV in PD but also highlights the capability of phytopharmaceutical active ingredients to be targeted toward stimulating NSCs differentiation. This cell therapy-based approach provides valuable insights for the treatment of PD and other neurodegenerative diseases.

## Figures and Tables

**Table 1:** Primer sequence used in qRT-PCR analysis.

Genes	Forward primer	Reverse primer	Accession numbers
Tubb3	CCAGATAGGGGCCAAGTTCT	GAGTCCCCCACATAGTTGCC	NM_139254.2
Nr4a2	AATCACTCGGCTGAAGCCAT	CTGTAGCTCTGAGAAGCGGG	NM_019328.3
SHH	AAGTATGGCATGCTGGCTCG	GCCACGGAGTTCTCTGCTTTC	NM_017221.1
Actb	CGCGAGTACAACCTTCTTGC	CGTCATCCATGGCGAACTGG	NM_031144.3
TH	ACCGAGAGGACAGCATCC	CACGGGCGGACAGTAGA	NM_012740.4
PCNA	CCACCATGTTTGAGGCA	CGAATCCATGCTCTGTAGG	NM_022381.3
Gapdh	TCTCTGCTCCTCCCTGTTC	ACACCGACCTTCACCATCT	NM_017008.4

## Data Availability

The data used to support the findings of this study are available from the corresponding author upon request.
